# Use of Human Intestinal Enteroids to Detect  Human Norovirus Infectivity

**DOI:** 10.3201/eid2509.190205

**Published:** 2019-09

**Authors:** Martin Chi-Wai Chan, Sarah K.C. Cheung, Kirran N. Mohammad, Jenny C.M. Chan, Mary K. Estes, Paul K.S. Chan

**Affiliations:** The Chinese University of Hong Kong, Hong Kong, China (M.C.-W. Chan, S.K.C. Cheung, K.N. Mohammad, J.C.M. Chan, P.K.S. Chan);; Baylor College of Medicine, Houston, Texas, USA (M.K. Estes)

**Keywords:** Cycle threshold, Ct cutoff, enteroids, infectivity, human norovirus, rRT-PCR, real-time reverse transcription PCR, viruses, enteric infections, Hong Kong

## Abstract

Tools to detect human norovirus infectivity have been lacking. Using human intestinal enteroid cultures inoculated with GII.Pe-GII.4 Sydney–infected fecal samples, we determined that a real-time reverse transcription PCR cycle threshold cutoff of 30 may indicate infectious norovirus. This finding could be used to help guide infection control.

Human norovirus accounts for 18% of acute gastroenteritis cases worldwide (*1*). Molecular nucleic acid tests, such as real-time reverse transcription PCR (rRT-PCR), are widely used for laboratory diagnosis of norovirus RNA in clinical samples (*2*). These molecular assays are virus specific and their analytical sensitivity is high, but they cannot distinguish between infectious and noninfectious viruses. To study the correlation between viral load measured by rRT-PCR and virus infectivity, we used a recently developed human intestinal enteroid (HIE) culture system (cultures that contain multiple intestinal epithelial cell types) for human norovirus. Ethics approval for this study was obtained from the Joint Chinese University of Hong Kong–New Territories East Cluster Clinical Research Ethics Committee (reference no. 2016.516).

## The Study

We examined the infectivity of human pandemic norovirus genogroup II genotype 4 (GII.Pe-GII.4 Sydney) strains at different inoculating levels by using the adult stem cell–derived HIE line J2 as described previously (*3*). We seeded enteroid monolayers on 96-well cell culture plates at a density of 6–8 × 10^4^ cells/well and maintained them in differentiation media for 5 days before being inoculated with norovirus. We used fecal samples from 3 norovirus-positive children and from adults in our norovirus surveillance program in Hong Kong (*4*). We prepared 1% fecal suspensions and filtered them once by using 0.22-μm centrifugal filters, then prepared 3-fold serial dilutions of fecal filtrates in infection medium and stored them at −70°C in multiple aliquots for single use. These dilutions mimicked a broad range of cycle threshold (C_t_) values of a widely used diagnostic rRT-PCR to represent high to very low norovirus levels (*5*). We used 33 μL of each dilution of fecal filtrate (brought to 100 μL in infection medium) for inoculation of each dilution and performed all virus inoculation steps and downstream cell cultures on enteroids with <20 passages in the presence of bile acid (glycochenodeoxycholic acid). We measured norovirus RNA levels in supernatant at 1, 24, and 72 h after inoculation by using rRT-PCR with a 10-fold serially diluted standard of in vitro–transcribed norovirus RNA. We considered a >10-fold increase in RNA level at 72 h after inoculation from baseline (1 h after inoculation) to indicate productive viral replication and to confirm the presence of infectious virus. We also subjected fecal filtrate dilutions to norovirus antigen detection by use of the Food and Drug Administration–cleared commercial RIDASCREEN Norovirus 3rd Generation EIA (R-Biopharm AG, https://clinical.r-biopharm.com), according to the manufacturer’s instructions. We used the same amount of fecal filtrate for inoculation into HIE and EIA measurement and compared the analytical sensitivity of HIE and EIA. 

**Figure 1:**
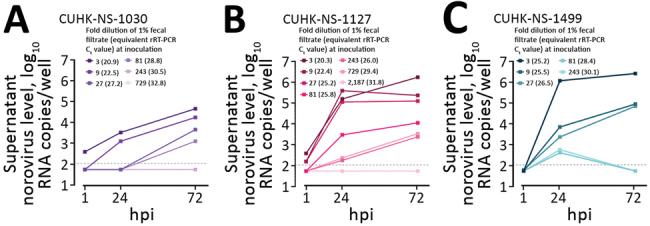
Performance of a human intestinal enteroid (HIE) line and an antigen-based enzyme immunoassay (EIA) to detect human norovirus in clinical fecal samples from 3 patients in Hong Kong. Replication kinetics of 3 human pandemic norovirus genogroup II genotype 4 (GII.Pe-GII.4 Sydney) strains (CUHK-NS-1030, from a 1-year-old boy; CUHK-NS-1127, from a 79-year-old man; CUHK-NS-1499, from a 46-year-old man) were tested in a monolayer culture of the adult stem cell–derived HIE line J2. We used 3-fold serial dilutions of norovirus-containing fecal filtrates to challenge J2 seeded on 96-well cell culture plates. Norovirus RNA levels in the culture supernatant at the indicated times were measured by rRT-PCR by using a 10-fold serially diluted standard of in vitro–transcribed norovirus RNA of genotype GII.Pe-GII.4 Sydney. Horizontal dotted lines denote the lower limit of detection of the rRT-PCR (13.8 RNA copies/reaction or 110 RNA copies/well) as determined by probit analysis. Samples with undetectable norovirus RNA were arbitrarily assigned a value equal to half of the lower limit of detection for calculation purpose. To convert the unit from RNA copies/well to RNA copies/mL, multiply the value by a factor of 10. hpi, hours postinoculation; rRT-PCR, real-time reverse transcription PCR.

**Figure 2:**
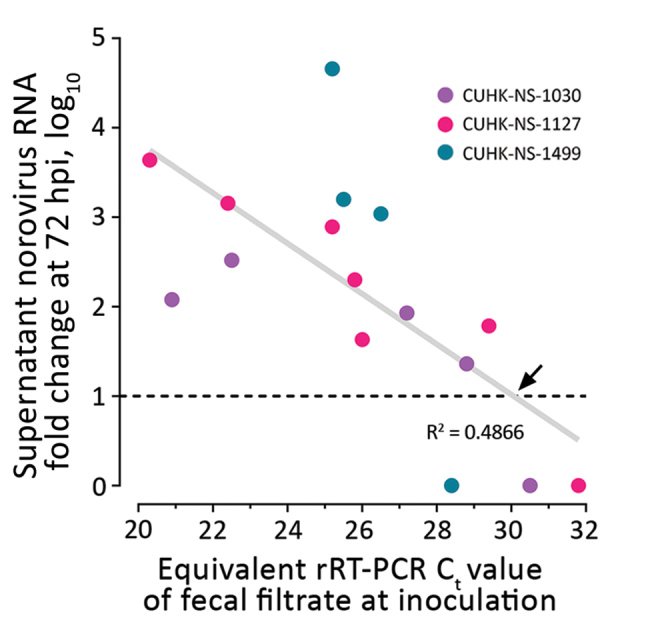
Estimation of a cutoff for rRT-PCR C_t_ value of inoculating fecal filtrate indicative of the ability to generate productive norovirus replication (i.e., containing infectious norovirus) in a human intestinal enteroid (HIE) line. We tested 3 strains of pandemic human norovirus genogroup II genotype 4 (GII.Pe-GII.4 Sydney) (CUHK-NS-1030, from a 1-year-old boy; CUHK-NS-1127, from a 79-year-old man; CUHK-NS-1499, from a 46-year-old man). We used 3-fold serial dilutions of norovirus-containing fecal filtrates to inoculate a monolayer culture of the adult stem cell–derived HIE line J2 seeded on 96-well cell culture plates. Productive norovirus replication was defined as having a supernatant viral RNA level increase of >10-fold at 72 hpi from baseline (1 hpi) (horizontal dotted line). RNA fold change data were derived from those shown in Figure 1 and were arbitrarily defined as 1 for those without observable viral RNA replication. For each strain, only the first dilution that resulted in undetectable viral RNA is shown and was included in downstream data analysis. The diagonal gray line denotes the best-fit line from linear regression, and the equation is *Y* = −0.2818*X* + 9.47, where *Y* represents RNA fold change and *X* represents C_t_ value. The black arrow specifies a C_f_ cutoff of 30.1 of inoculating fecal filtrate that yields productive norovirus replication as estimated from regression analysis. C_t_, cycle threshold; CUHK, Chinese University of Hong Kong; hpi, hours postinoculation; rRT-PCR, real-time reverse transcription PCR.

We selected 3 strains of norovirus GII.Pe-GII.4 Sydney, and all replicated productively in HIE line J2; the maximum fold increase of norovirus RNA ranged from 120 (2.1 log_10_) to 45,793 (4.7 log_10_). Higher levels of replication were obtained from fecal samples from adults (Figure 1). We identified no virus replication when cells were inoculated with fecal filtrate dilutions with C_t_ >30. The C_t_ values of the inoculating virus dilution that exhibited a transition from a positive-to-negative enteroid culture result (i.e., having a 10-fold RNA increase) were 27.7, 29.0, and 30.0 for each of the 3 strains. By using linear regression analysis on pooled data of the 3 strains, we estimated that a C_t_ cutoff of <30.1 in inoculating fecal filtrates would indicate the ability to generate productive norovirus GII.Pe-GII.4 Sydney replication (i.e., containing infectious norovirus) (Figure 2). 

From 2014 through 2018, a total of 114 (6.5%) of 1,754 norovirus-positive fecal samples from patients admitted to the Prince of Wales Hospital, Sha Tin, Hong Kong, with acute gastroenteritis had C_t_ values >30 (C_t_ median 17.8; interquartile range 14.8–22.3; range 5.5–39.2). Among the 1,579 (90.0%) genotyped samples, the proportion of GII.4 was 49.1%; other GII, 44.8%; GI, 5.2%; and co-infections with >1 norovirus capsid genotype, 0.9%. Analytical sensitivity of virus replication in HIEs for measuring moderate norovirus shedding was higher than that of EIA by being able to detect infectious virus in fecal filtrate dilutions with C_t_ values of 25–30 (Table).

**Table:** Comparison of rRT-PCR, HIE, and EIA used to detect human norovirus in samples from 3 patients in Hong Kong*

Isolate†	Fold dilution of 1% fecal filtrate	rRT-PCR C_t_	HIE (fold change at 72 hpi)	EIA
CUHK-NS-1030	3	20.9	+ (120)	+
	9	22.5	+ (331)	+/–
	27	27.2	+ (85)	–
	81	28.8	+ (23)	–
	243	30.5	– (ND)	–
	729	32.8	– (ND)	–
CUHK-NS-1127	3	20.3	+ (4,362)	+
	9	22.4	+ (1,436)	+
	27	25.2	+ (781)	+
	81	25.8	+ (200)	–
	243	26.0	+ (43)	–
	729	29.4	+ (61)	–
	2,187	31.8	– (ND)	–
CUHK-NS-1499	3	25.2	+ (45,793)	+
	9	25.5	+ (1,587)	+
	27	26.5	+ (1,094)	–
	81	28.4	– (ND)	–
	243	30.1	– (ND)	–

*Results for HIE line J2 and the antigen-based RIDASCREEN Norovirus 3rd Generation EIA (R-Biopharm AG, https://clinical.r-biopharm.com) were compared with the rRT-PCR that had been widely used as a standard for clinical diagnostics of norovirus infections. The same amount of fecal filtrate was used in HIE line J2 inoculation and EIA measurement at the indicated dilutions. In HIE, numbers indicated fold changes of norovirus RNA level in culture supernatant at 72 hpi from baseline (1 hpi); an increase of >10-fold was used to confirm the presence of infectious norovirus. Ct, cycle threshold; CUHK, Chinese University of Hong Kong; EIA, enzyme immunoassay; HIE, human intestinal enteroid; hpi, hours postinoculation; ND, not detectable; rRT-PCR, real-time reverse transcription PCR; +, positive as defined for each assay; –, negative as defined for each assay; +/–, equivocal as defined according to the manufacturer’s instructions of the EIA.

†CUHK-NS-1030, from a 1-year-old boy; CUHK-NS-1127, from a 79-year-old man; CUHK-NS-1499, from a 46-year-old man.

## Conclusions

The limitations of highly sensitive molecular nucleic acid tests and the clinical value of proving virus infectivity were demonstrated in a recent study of Zika virus infection that found that the virus could be detected by culturing samples with high viral RNA levels only (*6*). For human norovirus, laboratory tools to detect its infectivity have been lacking. Recent technologic advancement in culturing human norovirus in HIE provides a chance to examine norovirus infectivity in laboratory settings (*3*). Using pandemic norovirus GII.Pe-GII.4 Sydney strains, we showed that the most sensitive method for detecting human norovirus was rRT-PCR, followed by HIE infection; EIA was the least sensitive. We experimentally determined that norovirus inocula with C_t_ <30 can robustly yield productive virus replication in HIE, suggesting the presence of infectious virus. This value concurs with findings of a study that proposed an optimal C_t_ cutoff of 31 when attributing a disease to norovirus by comparing between symptomatic and asymptomatic cases (*7*). Our findings imply that a small proportion (≈6.5%) of patients shedding low levels of norovirus RNA (C_t_ >30) may not be infectious.

In an experimental human volunteer challenge study with norovirus GII.4, low levels of viral RNA shedding were found 10 days after challenge (*8*). HIE infection can now be used to reevaluate archived samples to better define parameters that would correlate with the infectious period of norovirus gastroenteritis to guide infection control. Of note, in a meta-analysis, the prevalence of norovirus RNA shedding from asymptomatic patients was estimated to be ≈9% (*9*). Moreover, in a large-scale study of outbreak data from CaliciNet (https://www.cdc.gov/norovirus/reporting/calicinet/index.html), a national norovirus surveillance network in the United States, the median C_t_ value among asymptomatic patients was 28 (*10*). We hypothesize that a substantial proportion of asymptomatic patients are shedding infectious norovirus and that their role in spreading the virus merits our attention. We have shown that high levels of norovirus replication can be achieved from fecal samples of adults, not just young children, in HIE cultures (*11*). 

The use of a fixed C_t_ cutoff in clinical context needs to be interpreted with caution. First, neither a standardized protocol to perform rRT-PCRs nor a World Health Organization International Standard for norovirus is available to harmonize assay variability across laboratories worldwide. Differences in recovery among nucleic acid extraction methods may further complicate reproducible determination of C_t_ values. Second, virus culture systems in cell lines generally lack sensitivity (*12*), and that of HIE remains unknown. However, it is probably not optimal because input genome equivalents for norovirus to achieve replication are not extremely low (50% infectious dose 4.4 × 10^2^ to 2.1 × 10^3^ copies/well) (*3**,**11*). It is possible that samples with C_t_ >30 might still contain infectious virus and that low amounts of replicating norovirus would only be detected with further serial propagation of the virus. Third, norovirus replication varies between samples and virus genotypes in HIE culture (*11*).

In summary, we demonstrate that a C_t_ cutoff of 30 for a widely used clinical diagnostic rRT-PCR can indicate the presence of infectious GII.Pe-GII.4 Sydney norovirus in an HIE culture model. Patients shedding low levels of norovirus RNA may not be infectious, which should be considered both for estimation of attributable norovirus burden and for clinical management of viral gastroenteritis.
